# Combining Task and Motion Planning: Challenges and Guidelines

**DOI:** 10.3389/frobt.2021.637888

**Published:** 2021-05-19

**Authors:** Masoumeh Mansouri, Federico Pecora, Peter Schüller

**Affiliations:** ^1^ Intelligent Robotics Lab, School of Computer Science, University of Birmingham, Birmingham, United Kingdom; ^2^ Multi-Robot Planning and Control Lab, Center for Applied Autonomous Sensor Systems, Örebro University, Örebro, Sweden; ^3^ Knowledge-Based Systems Group, TU Wien, Vienna, Austria

**Keywords:** task and motion planning, integrative AI, knowledge representation, automated reasoning, industrial applications of robotics

## Abstract

Combined Task and Motion Planning (TAMP) is an area where no one-fits-all solution can exist. Many aspects of the domain, as well as operational requirements, have an effect on how algorithms and representations are designed. Frequently, trade-offs have to be made to build a system that is effective. We propose five research questions that we believe need to be answered to solve real-world problems that involve combined TAMP. We show which decisions and trade-offs should be made with respect to these research questions, and illustrate these on examples of existing application domains. By doing so, this article aims to provide a guideline for designing combined TAMP solutions that are adequate and effective in the target scenario.

## 1 Introduction

**FIGURE 1:**
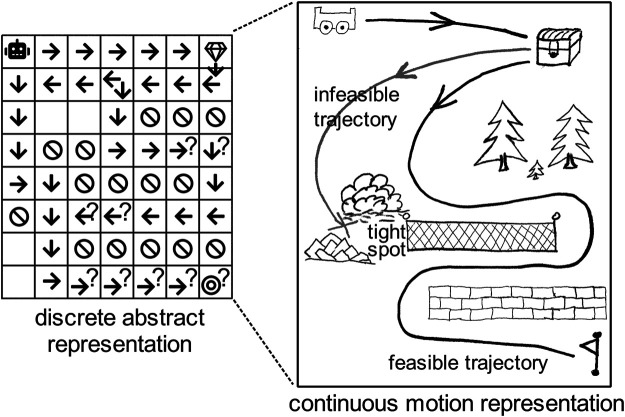
Example of combining TAMP using an abstract and a motion representation with uncertainty (question marks) and two abstract solutions of which one is infeasible due to a tight spot.

This paper addresses a known problem in planning for robots, namely, that of combining Task And Motion Planning (TAMP). As robots have been increasingly deployed in challenging, unstructured object- and interaction-rich environments, combined TAMP has received extensive attention from the robotics community. Examples include TAMP solutions for autonomous excavators pushing gravel in construction sites, or autonomous mining robots drilling the ground to extract materials. To make a robot operate competently in such environments, researchers have combined methods from different sub-fields of Artificial Intelligence, including task planning to compute appropriate actions, motion planning to generate motions using geometric models, and control to compute feasible trajectories. The numerous efforts dedicated to combining task and motion planning highlight a common scientific challenge, namely, that the level of abstraction varies across planning models: discrete domain representations for task planning and continuous models for motion planning and control. Figure 1 illustrates several challenges that commonly occur in combining TAMP: finding solutions in discrete search spaces that are infeasible in continuous space, abstraction of continuous space, and uncertainty (e.g., due to obstructed view). Currently, solutions to combined TAMP vary in the way in which they explore the (joint) continuous-discrete search space.

When designing a TAMP method, the application requirements pose scientific questions beyond how we explore the search space. This paper attempts to lay down relevant challenges and research questions that can be used as guidelines for solving real-world TAMP problems. We consider relevance with respect to two criteria: 1) addressing the challenge is technically difficult and requires significant research and innovation; 2) advances in addressing the challenge have a large impact in current industrial applications. These research questions are the following:

•*Q1: How can a domain be divided into multiple levels of abstraction, and what are effective methods for finding a globally feasible solution that obeys all constraints in all abstraction levels?*
•*Q2: How should symbolic and continuous knowledge representations be reasoned upon jointly?*
•*Q3: What classes of methods exist for learning models, specifying models, and performing the two together? In particular what are the options for combining existing task and motion planning methods with machine learning?*
•*Q4: How to enable online decision making in combined task an motion planning? How can we guarantee the consistency of decision-making in such settings?*
•*Q5: Which methods should be used to deal with uncertain perception in combined task and motion planning? Should uncertainty be considered in one of the decision making processes or both?*

We will show how these five aspects reflect the major gaps/open questions in the current state of advancement in planning for robots. We will also show that these questions include the key choices that need to be made when combining task and motion planning in real applications. As our analysis makes evident, alternative solutions are proposed to similar questions. This often depends on aspects of the application context, and on the different assumptions and trade-offs that can be made. This paper serves as a guideline for navigating the landscape of existing solutions and their caveats when designing combined task and motion planning methods. As opposed to the latest survey on this topic (Garrett et al., 2020), we here analyze a wider scope of concerns within TAMP and their combination; in particular, we discuss several orthogonal aspects, including uncertainty handling and online planning. Also, the discussion is centered on five open research questions and how answering them matters in a selection of industrially-relevant application contexts.

## 2 Discussion Over Five Research Questions

Task and motion planning methods are categorised based on the different class of algorithms used in planning tasks and motions, as well as the way in which these two types of methods are combined. In their widely referenced textbook on Task Planning, Ghallab et al. (2016) organised their discussion based on available *representation and reasoning* choices in task planning for the purpose of acting. Similarly, for the motion planning reference book, LaValle (2006) centered his discussion around how a robot world (*via* a geometric and configuration space) can be *represented* and the efficient algorithms to explore those spaces (*reasoning* about motion). Our Q1 concerns the representational choices that need to be made combining these two types of representations, each belonging to a different level of abstraction. Q2 follows the logical next step of addressing issues related to reasoning about these representations in combination. Q3 discusses using learning algorithms in lieu or in support of both representation and reasoning. Q4 concerns applications where TAMP techniques should be integrated online with acting, and Q5 addresses the all-important question in robotics of planning with uncertain knowledge. There are, of course, other open issues in combined TAMP — however, we believe that these are either subsumed by these questions (e.g., how to discretize the environment for task planning), or are of lesser general interest because they are relevant to very specific application settings. We also claim that it is not possible to answer any of the four questions above in isolation. The issues around representation, reasoning, how we obtain a joint model, online reasoning, and uncertain or incomplete knowledge of an environment, are all interdependent. For example, consider an application where we have a TAMP problem that should be solved in an online manner. For the TAMP solution to be appropriate for this purpose (addressing Q4), the chosen representation for the task and motion (addressing Q1) should be computationally adequate for enabling fast joint reasoning (addressing Q2). In Section 4, we provide four applications requiring combined TAMP and discuss a possible order in which these questions can be addressed.

## 3 Analysis of the State-of-the-Art

We categorise the existing TAMP methods around our five research questions. Note that the categorisation is not crisp, i.e., one paper can belong to more than one category.

### 3.1 Abstraction

In this section, we mainly discuss how different levels of abstractions interact by means of a shared abstraction (partially addressing Q1), and leave the discussion about choice of knowledge representations at each level to the next section. A shared abstraction must have the capacity to represent knowledge at different levels. Shared abstractions can be realized by the mechanism of an already existing logic [e.g., Satisfiability Modulo Theories (SMT) (Nieuwenhuis et al., 2006)], various forms of constraint-based approaches (e.g., meta-constraint reasoning), or a novel formalism designed specifically for enabling interaction between these levels.

SMT is built upon the notion of augmenting the Boolean Satisfiability Problem (SAT) with the ability to reason about several diverse background theories. For instance, Nedunuri et al. (2014) encode high-level robot requirements in a SAT formulation, and the background theories are linear arithmetic and functions which relate to the physical configuration of the robot and objects in the environment. Another example of reasoning with a shared abstraction is meta constraint reasoning. In this problem formulation, task and motion planning problems are modeled as different instances of Constraint Satisfaction Problems (CSPs) at different levels of abstraction. So-called meta-constraints capture the dependencies between task and motion CSPs (Mansouri, 2016). Instead of adapting known knowledge representation like SMT or CSP, Dantam et al. (2018) propose a flexible framework that employs a uniform interface (called scene graph) as a shared abstraction to connect motion and environment models with task states. Similarly, Gaschler et al. (2013) treats volumes as a shared abstraction.

In addition to shared representations, formal methods are used to provide behavioral guarantees at all levels of abstraction. In these methods, formal synthesis provides a framework for specifying tasks in a mathematically precise language, and automatically transforming these specifications into correct-by-construction robot controllers (Kress-Gazit et al., 2018). Linear Temporal Logic (LTL) is a formal language that is commonly used in TAMP formulations (e.g., Plaku, 2012). Also, Signal Temporal Logic (STL) also exist to relate logic predicates to continuous-time signals (e.g., Maler and Nickovic, 2004).

The TAMP domains in the instances described above were divided based on the capacity of the shared knowledge representations which ensure to find global feasible solutions for all levels of abstraction. Choosing a shared representation capable of maintaining such global consistency is an effective method to divide an overall TAMP problem to a set of sub-problems embedded in a shared representation. In the following section, we discuss other ways to enable interactions between levels of abstraction.

### 3.2 Symbolic Versus Continuous Models

In TAMP, symbolic knowledge representations are often relevant in most variants of task planning; by contrast, most models that are relevant for motion planning are expressed in terms of variables with continuous domains. Furthermore, different types of models (and, hence, different forms of automated planning) may be relevant in a given application, e.g., continuous time and events, metric maps and qualitative spatial relations, kinodynamic motion models and symbolic preconditions for acting. In the following, we analyze various approaches for combining symbolic and continuous models.

To enable joint reasoning across symbolic and continuous domains (addressing Q2), *Procedural Attachment* is a common approach. In procedural attachment, feasibility of actions in terms of kinematics, dynamics, and geometric constraints is assessed through a procedure, e.g., an external motion planner, that is attached to the symbol(s) representing that action at a high level of abstraction. The approaches differ in the reasoning techniques used for high level action and task planning, e.g., Boolean satisfiability (Havur et al., 2013), PDDL planning (Srivastava et al., 2014), Hierarchical Task Network (HTN) planning (Kaelbling and Lozano-Pérez, 2011), or Answer Set Programming (ASP) (Erdem et al., 2016); as well as the attached procedures, e.g., simulation-based verification (Mosenlechner and Beetz, 2011), geometric reasoning (Lagriffoul et al., 2012) or motion planning (Havur et al., 2013). Another common approach to enable joint reasoning is to use sampling-based methods, where both task and motion solutions are combined in one common space for a probabilistic search to navigate in. Such methods can use conditional samplers that are provided as part of a domain specification, hence domain knowledge improves sampling in the (usually large) solution spaces (Garrett et al., 2018).

Sampling-based methods incorporate discrete sampling for task planning into the (usually non-discrete) sampling process used in many approaches to motion planning. Procedural attachment is exactly the opposite strategy: motion planning is attached to certain logical predicates that are processed by the algorithm of the task planner. A notable difference is that motion planning methods are directly used as sub-procedures in procedural attachment, while sampling-based methods do not use a task planner; they recast task planning as sampling. Therefore, procedural attachment and sampling-based methods are two extremes in a potential continuum of integrating task and motion planning along the aspect of ‘what is the leading formalism’ of the integration — the task level or the motion planning level.

Historically, combining task and motion planning *via* procedural attachment has been very successful in promoting the role of Symbolic AI reasoning in robotics. However, procedural attachment fails to provide a scalable, general technique for integrating very diverse forms of reasoning. There are reasons behind this shortcoming. One is that procedural attachments often do not capture inter-dependencies among sub-problems, i.e., each sub-problem solver is not aware of requirements of the domain that pertain to other sub-problems. These approaches lack a clear and transparent means to specify inter-dependencies between sub-problems; this is due to the fact that such a specification would have to combine notions/concepts that are expressed in different KR formalisms. Finally, note that some flavors of procedural attachment permit limited inter-dependencies between low-level sub-problems, e.g., HEX-programs (Erdem et al., 2016).

Regarding integration of symbolic and continuous reasoning, several properties of the application area need to be considered. if it is sufficient to find solutions that are close to the optimal, sampling-based methods are a better choice. However, procedural attachment or more powerful symbolic task-level methods are preferable for cases when the task level search is highly complex so that only few solutions exist, and sampling would potentially yield no solution. Procedural attachment can also be used when low-level reasoning itself is required to be split into many small sub-problems so that can be solved independently.

### 3.3 Specifying Versus Learning

Q1 and Q2 discussed so far concerned the choices TAMP methods make regarding the representation of planning models. Q3, on the other hand, has to do with how to obtain these models, and is particularly in focus today thanks to the rise in popularity of machine learning techniques, in particular deep (reinforcement) learning.

The recent AlphaGo breakthrough has had a great impact in many areas of AI, including TAMP. Kim et al. (2019a) propose an actor-critic algorithm that learns from planning experience to guide a planner. They have also investigated how to predict global constraints on the solution for generic TAMP problems using a scoring function to represent planning problem instances (Kim et al., 2019b), and have developed an algorithm that learns a stochastic policy from past search trees using generative adversarial nets, for problems with fixed numbers of objects (Kim et al., 2018).

Using a learning method for planning does not originate form AlphaGo. In a review paper from 2012 (Jiménez et al., 2012), learning for planning is described on the task level and with respect to discrete planning actions and states; the purpose of learning is to acquire knowledge about 1) action conditions and effects, i.e., the domain; or 2) heuristic knowledge for guiding the search process faster to a goal state. A later review from 2018 (Arora et al., 2018) focuses only on 1) and refers to the integration of task-level and motion planning as part of a list of “guidelines that a robotic system can follow in order to be proclaimed autonomous”. This review also mentions Surprise Based Learning (SBL) (Ranasinghe and Shen, 2008), which learns a domain description from execution monitoring and interleaves prediction of future states and monitoring of actually reached states to improve that domain theory. SBL is the main work that embraces the idea of an incorrect domain theory. We argue that Motion Planning also embraces that idea but in an orthogonal way: TAMP is based on the use of a domain description which is correct on its level of abstraction and coarse enough to permit efficient planning; however, due to its coarseness, planning with this domain description requires an integration with motion planning to ensure that the task plan can be realized in the concrete domain. Contrary to SBL, TAMP does not attempt to repair the task-level domain description, because any repair that would ensure full correctness would at the same time make the domain description useless for efficient planning algorithms.

Balac et al. (2000) employs regression tree learning to predict the influence of terrain on the efficiency of high-level actions to be used in the task planner. In other words, their system performs learning of low-level action cost to be used as a parameter in the high-level domain for task and motion planning. In a similar vein, imitation learning has been used to learn motion primitives corresponding to a manually specified high-level task structure from one-shot demonstrations of a human (kinesthetic teaching) who also gives verbal cues about the task at hand (Caccavale et al., 2019). An attention mechanism automatically segments motion tracking data from the human and assigns recorded motion primitives to sub-tasks. In general, learning is a better choice for designing levels of abstraction within TAMP such that the specification is difficult to achieve or imprecise due to the complexity of the domain or lack of knowledge on the environment. We will see concrete examples of learning parts of domains in our illustrated use cases in Section 4.

### 3.4 Online Planning

In real-world applications, automated planning systems are often required to make decisions online, while previous plans are already under execution. When planning for motions, this is known as Receding Horizon Control, or Model Predictive Control. In task planning, methods ensuring the ability to update plans online is often referred to as continuous planning. Moreover, incomplete knowledge about the domain requires assumptions to be made for planning, and in online planning these assumptions may be revised multiple times during physical execution of the plan. This concern can be summarised as in Q4, around which we analyze the current methods.

Many approaches to online task and motion planning can also be relevant to *plan-based robot control* (see the next subsection). Online planning is about radical changes of plans due to contingencies, whereas control is more about small disturbances that the controller can compensate in its local environment without affecting the overall plan. Also, we assume that a plan obtained by an online planner is only preliminary until it has been executed. This is because the environment is highly non-deterministic, described probabilistically, partially unknown, or a considerable fraction of actions is bound to fail. As a case in point, a human-robot collaborative manipulation system has to adapt its cooperative behavior during execution due to the continuous human intervention (Cacace et al., 2018).

Contingency planning methods do not compute plans in an online fashion, rather prepare them for dealing with foreseeable failures. The way these planners work is to put in sensing and repair actions in an original plan, sometimes conditionally, where certain action failures are likely to happen. In this way, contingency planners ‘program’ replanning already into the initial plan. An example is the HCP-ASP hybrid conditional planner, where conditional actuation and sensing actions are modeled in ASP (Yalciner et al., 2017). An ASP solver computes feasible branches of a conditional plan using external atoms that account for continuous feasibility checks (e.g., collision checks).

In summary, with respect to online planning we have to first identify which classes of possible action failures or modified environment situations we want the robot to be robust against. It is important to determine in the beginning whether the goal of the plan is subject to change. Also, it is important to know how fast new knowledge needs to be integrated into the plan, in other words, how long can we afford to execute the ‘old’ plan before knowing and switching to the ‘new’ plan.

### 3.5 Planning With Uncertainty

Robots epitomize the need for automated planning methods, which provide them with the means to achieve goals. Yet the physical nature of robot systems, as well as the uncertainty connected to robot behaviors and perception, destroy many of the assumptions made by current methods for planning. Q5 focuses on this aspect of TAMP.

Many pioneers in using automated task planning for deriving robot behaviors use the term plan-based robot control to distinguish planning for robots from planning for other systems. The focus here has been on aligning the belief-state of the robot with the symbolic planning process. The latter can employ one of the many approaches to task planning, from Hierarchical Task Networks for TAMPs in partially observable environments (e.g., Weser et al., 2010) to inferring the most appropriate plan from a pre-defined plan library using probabilistic representations (e.g., Beetz et al., 2001). Decision-theoretic task planning methods, and specifically Markov Decision Processes (MDPs) and Partially Observable Markov Decision Processes (POMDPs) are the most prevalent approaches for tackling various types of uncertainty in TAMP formulations (e.g., Şucan and Kavraki, 2012; Kaelbling and Lozano-Pérez, 2013; Hadfield-Menell et al., 2015). A recent work includes an anytime algorithm of TAMP generating policies for handling multiple execution-time contingencies using MDP-based modeling of actions which corresponds to an infinite set of motion planning problems (Shah et al., 2020).

A reactive type of formal methods has also been used to provide behavioral guarantees in typically uncertain or adversarial environments [e.g., within a receding horizon paradigm (Raman et al., 2015)]. LTL-based abstractions can be used to account for delays and measurement errors as a form of uncertainty modeling in TAMP (Liu and Ozay, 2016). Also, recent theoretical advancement in synthesis methods for uncertain MDPs that provide better noise modeling than classical MDPs, have shown promise for uncertainty handling in TAMP (e.g., Lahijanian et al., 2015).

With respect to uncertainty, it is essential to identify whether uncertainties on the task level can be foreseen so that we can create robust plans regarding the uncertainty models. For uncertainties on the motion-planning level, we have to validate whether it is sufficient to use local control methods to tackle uncertainties, or there is a need for deferring to the task level. In general, the more uncertainty we have to deal with, the more likely it is that some type of Online Planning (see previous section) is to be necessary.

## 4 Applications

Now that we have outlined the key questions underlying the realization of combined TAMP, we illustrate how some of these aspects have been considered in concrete industrial applications. There are many industries where combined TAMP is required for long-term autonomy. These range from automation of heavy-duty machines operating in unstructured environments such as mines or construction sites, to that of robots in controlled and unobstructed environments such as factories. The challenges these domains bring about differ in nature, but fall in the range of the questions we have outlined above. Specifically, we identify three broad issues posed by such industrial applications.

First, in some domains, tasks carried out by the robot(s) in the environment affect the motions that can be carried out in realizing other tasks. This is true in mining, for instance, where operations like drilling have a permanent effect on navigability. In general, decisions over the order of the tasks not only affect the subsequent motions but also the environment. We illustrate an example of such applications in Section 4.1.

Second, an industrial application has crucial qualitative requirements to guarantee safe operation, e.g., there should always be a machine in a certain station whenever one is in another station. These seemingly simple constraints have ramifications beyond the task level, as they affect all levels of abstraction including the low-level control. For instance, a machine may need to accelerate to fill the place of another machine leaving an active station. We will examine such an instance of TAMP in an application of electric haulers in a quarry in Section 4.2.

Third, peculiarities of industrial applications and their consequences in their TAMP formulations are not limited to those that derive from unstructured, outdoor environments. Even in more structured environments, like factories, a relevant issue is how to design the environment for efficient task and motion planning. In manufacturing, for instance, motion planning can often be greatly simplified at the cost of limiting the flexibility of the robotic solution. We address the relation between specifications for task planning and their implication on the resulting TAMP formulation in Section 4.3.

Fourth, qualitative specifications may be relevant to ensure task achievement under extreme uncertainty. This is case in mission planning for Autonomous Underwater Vehicles, where task plans and motions are affected by currents and other complex environmental phenomena that are difficult to model. We discuss the use of learning predictive models for use in combined TAMP in Section 4.4.

Although these problems are relevant in many more applications than those cited below, we have made a selection of few concrete examples in each of these three categories in order to underscore the impact that innovation in task and motion planning can have in the real world. We conclude the section by indicating some good practices derived from these examples.

### 4.1 Drill Planning for Open-Pit Mines

In this section we analyze the *drill planning* problem within an application involving a fleet of surface drill rigs operating in a common area of an open-pit mine, called a bench.

#### 4.1.1 Problem and requirements.

A set of drill targets in a bench is given; at each target, a blast hole is to be drilled and filled with explosive material, which will then be detonated to produce rubble that will be processed into ore. The drill planning problem consists of computing a plan that involves machines reaching each drill target in a bench and performing the necessary operations to drill the blast hole. Drilling produces piles of excess material around the hole. These piles constitute obstacles for the machine itself and other machines, hence no machine can drive over them. A solution to the drill planning problem should take into account the emerging obstacles as well as all other common TAMP requirements (e.g., avoiding machine-machine collisions) to be executable by the drill rigs.

#### 4.1.2 Integration Challenges

The drill planning problem can be seen as a combination of several sub-problems: task planning (consisting of deciding the sequencing of the targets to be drilled), motion planning, and coordination. These problems cannot be treated separately, as the solutions of each problem depend on each other. For instance, task planning must lead to a sequence of drill targets that accounts for the piles generated after drilling (which become obstacles that must be taken into account in motion planning). In other words, the order in which the targets are drilled will affect the ability of the machine itself and other machines to traverse on the bench. Hence, it is necessary to subject the possible choices made to solve one problem to the choices made in resolving the other problems, e.g., verifying through motion planning that a chosen sequence of targets to drill will be kinematically feasible and will avoid the piles of material produced by drilling. There are two approaches in the literature addressing the drill planning problem. One approach is based on meta constraint reasoning (Mansouri et al., 2016) in which the task planning, motion planning and coordination problems are modeled as CSPs at different levels of abstraction, and the meta-constraints capture inter-dependencies between the tasks and motions as a shared abstraction. The second approach implements a multi-abstraction search where an abstract solution is refined incrementally with different types of search at different levels of abstraction (Mansouri et al., 2017).

#### 4.1.3 Considerations

This application allows us to make several statements pertaining to questions Q1– Q3.

Q1: One common way to deal with multiple levels of abstraction is to abstract away the continuous (geometric) representation – the motions and the piles in this problem – in order to obtain a fully discrete (graph) representation. The graph representation of the drill planning problem forms a variant of the Traveling Salesperson Problem (TSP) where nodes are the drill targets, and edges represent abstracted motion between the nodes. Then, the problem is to find a shortest closed path (tour) in the graph such that every node is visited only once. In a TSP, regions to be visited are associated to nodes in a graph, and each node should be traversed exactly once. Roughly speaking, each region along a tour acts as an “obstacle” that appears dynamically once the node is visited, and which must be avoided while visiting other nodes. However, the TSP employs the abstract notion of a graph to represent locations and their connectivity, thus ignoring the geometrical extent of the locations. Ignoring the geometric reality of the nodes in the TSP, and the fact that paths between them are affected by this spatial extent, leads to solutions that may not be feasible in practice, as they ignore the further constraints to the motion space that derive from the drilling tasks. This points to a rather general observation: abstracting away certain aspects of the problem representation preserves correctness only if we do not lose information regarding the dependencies between different aspects of the problem (in this application, the geometrical extent of the drill targets). An alternative way is to keep each representation at its own level of abstraction, and to leverage a common language to combine relevant knowledge among different levels of abstraction. To enable the use of a common language, we should first identify sub-problems of the overall problem. Furthermore, we need to identify dedicated solvers, each of which focuses on a subset of aspects of the overall problem, e.g., a motion planner verifies kinematic feasibility and absence of collisions, while a scheduler verifies that coordination choices are temporally and spatially feasible. Validated solutions for each sub-problem can be see as *constraints* that account for particular aspects of the overall problem. As remarked below, constraints can play the role of a common language to facilitate joint reasoning.

Q2: Where we discard the continuous (geometric) representation of motion and piles under a fully discrete (graph) representation, we effectively disable joint reasoning for the drill planning problem. Nevertheless, one might solve a TSP over the graph representation as a proxy to solve the drill planning problem, and use a post-processing step to filter out TSP solutions that are infeasible with respect to motion and pile constraints. To enable joint reasoning in the second alternative of dealing with multiple levels of abstractions, we can use the common language, in this case constraints, in a common constraint network (Dechter, 2003) to model the search space of all problems jointly. In this way, each dedicated solver only operates in the relevant level of abstraction, and validating solutions to sub-problems is reduced to posting constraints in the common constraint network and verifying its consistency. This approach is realized in several different applications, including drill planning (Mansouri et al., 2016) and integrated task and motion planning for warehouse management (Mansouri et al., 2015).

Q3: Machine learning can be useful not only for generating planning models, but also to generate heuristics for efficiently exploring search spaces. In the drill planning problem, we can learn patterns from examples provided by human experts for sequencing decisions in regions where machines have limited space to manoeuvre (Mansouri et al., 2016). In general, learning from humans is important for uptake by the industry, as end users want machines to adhere to the best practices of humans while expending little effort in specification/knowledge engineering. We can also use clustering methods for analyzing the topology of a bench, which will allow to cluster targets into groups for which there are only few reasonable sequencing possibilities and that are easy to navigate in sequence. This again will alleviate the computational burden of finding sequences in a joint search space, which is strongly affected by constraints on motion (Mansouri et al., 2016; Mansouri et al., 2017).

### 4.2 Multi-Hauler Planning for Quarrying

In this section, we focus on an instance of TAMP for multiple electric haulers operating in a quarry. The important challenge in this application is to provide team-level guarantees over team behaviors in the presence of high uncertainty over the durations of navigation actions. The problem requirements presented in this application can be found in other real-world robotics applications, such as mining, construction, and warehouse automation.

#### 4.2.1 Problem and Requirements

A team of autonomous electric haulers transport material between stations in a quarry. At the unloading station, a hauler can unload gravel obtained from two crushers. The primary crusher (PC) constantly produces gravel, which is continuously output *via* a conveyor belt. The production of gravel at the PC cannot be stopped under normal circumstances, hence, there should *always be a robot under the PC* so that gravel does not accumulate on the ground, obstructing access to the PC and halting the entire process. The secondary crusher (SC) does not have this constraint, as the gravel produced there is loaded onto haulers manually. Also, robots are required to leave the PC when full. The aim is to maximize the throughput of the overall system, i.e., the amount of gravel dumped at the unloading point. The important constraint of this application is to guarantee that there is always a hauler under the PC. In this instance of TAMP, we require to solve task planning for the team-wide decisions of which robot visits which station in what order, and motion planning and coordination for the decisions of how, when and where the robots move. Henceforth, we refer to this instance as a *multi-hauler planning* problem.

#### 4.2.2 Integration Challenges

In order to respect the constraint of “there should always be a robot under the PC”, we have to able to compute exactly how long it takes for a hauler to go from one station to another station to make sure that we dispatch the robot in an appropriate time. Being too conservative and sending as many robots as available to the PC, makes the SC useless, and negatively affects the throughput by wasting robots being in a queue to reach the PC. However, the durations of navigation actions of the haulers in the quarry is very uncertain. This uncertainty stems from many sources, e.g., the dynamics of individual robots are typically only partially known; robots may navigate differently in different parts of the environment (e.g., skidding over a sandy patch of terrain, proceeding more slowly in the vicinity of pedestrians); task-dependent factors may affect how robots navigate (e.g., slow movement due to a heavy load); and interactions between robots jointly navigating in a shared space introduce further unmodelled dynamics (e.g., robots yielding to, or avoiding, each other). The multi-hauler planning problem was addressed in the literature by a hierarchical approach based on Generalised Stochastic Petri nets (GSPN) for modeling team behavior, where accurate probabilistic models of path durations are obtained *via* integration with a lower-level team controller (Mansouri et al., 2019). The GSPN is then interpreted as a MDP for which policies can be generated so that team performance is optimized whilst avoiding the exponential blow-up associated with the construction of a full joint model.

#### 4.2.3 Considerations

This application is most relevant to two of our original questions.

Q3: Today’s commercial solutions for multi-robot path planning remove many source of uncertainty by engineering the environment. Such assumptions are not applicable in many real-world applications including multi-hauler planning. For this reason, current industrial practice relies on fixed, hand-crafted policies for selecting tasks for robots and dispatching them to their destinations. We should instead replace the current practice with an automated planning system that does not make assumptions on the map, the robot geometries, the paths followed by robots, or their kinematics and dynamics. The system should provide a means to easily specify high-level requirements on team behavior, including safety constraints, and it should scale to realistically-sized teams. In order to robustly maintain the safety specification for the PC, models of navigation task duration can be learned. In the absence of real data due to the difficulties of deploying real experiments, we can learn from simulations of the team navigating in the target environment in this case a quarry (Mansouri et al., 2019). To explore the range of multi-robot navigation experiences relevant for the target environment, the robot team must operate in a way that is as similar to the desired behavior as possible. To achieve this, the team should be controlled in simulation using a controller which integrates coordination, motion planning and robot control (e.g., Pecora et al., 2018), and supports the injection of external navigation choices for robots. Given these choices, the controller generates multi-robot paths that take into account the kino-dynamic constraints of individual robots. These paths are jointly executed and supervised by the controller. When generating data for learning, a randomised policy can be used to provide navigation choices.

Q5: The main source of uncertainty in this problem is the duration of navigation actions. We require a method for multi-hauler planning that accounts for this uncertainty. A popular approach to planning with uncertainly is to use MDPs, where uncertainly is modeled in the outcomes of actions. However, it is not accurate to directly model the learned probabilistic model of duration as an action outcome in an MDP. Instead, we can use a stochastic extension to Petri nets to model team behaviors with probabilistic models of path durations. This can then yields an MDP which can be solved to generate policies that optimise team behavior against the team requirements and performance objective.

### 4.3 Assembly Planning for Industrial Manipulators

In this section, we analyze an application of dual-arm manipulators in assembly tasks.

#### 4.3.1 Problem and Requirements

**FIGURE 2:**
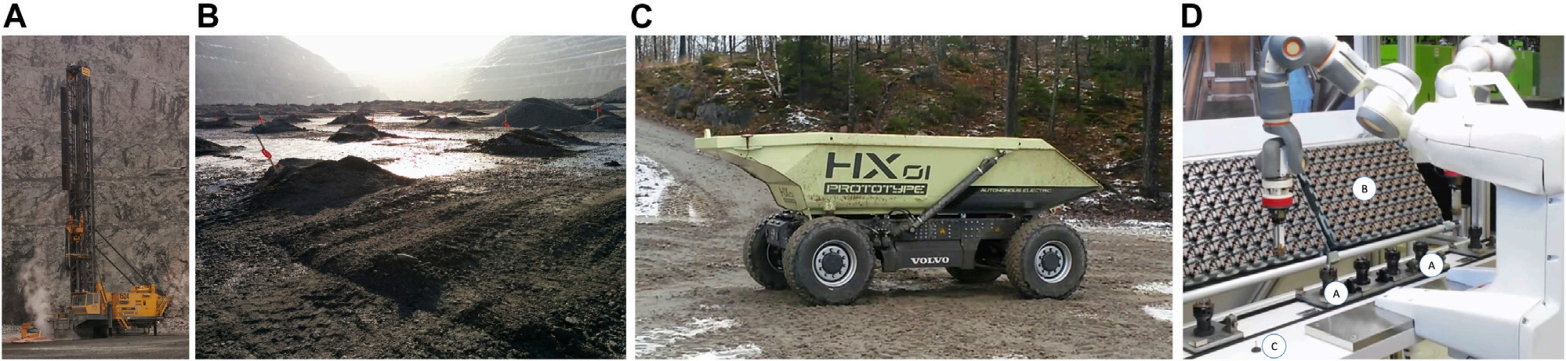
Drilling machines and the resulting holes in an open-pit mine **(a, b)**, autonomous construction machines in a quarry **(c)**; a dual-arm robot assembling wiper motors **(d)**.

A modern lightweight dual-arm robot, e.g., the ABB Yumi, is deployed to assemble pieces of wiper motors. The workstation is depicted in Figure 2C, where the rotors are already inserted into workpiece holders (A) on a conveyor system, arriving in groups of five. The stators with the brushes and the electric interfaces are supplied in transport containers (B). Mounting a stator on a rotor requires to place a cone-shaped tool on the motor shaft temporarily. (C) marks the position the robot picks up of a tool. Such flexible production requires fast methods to specify new tasks for these robots, and classical teach-in by means of fixed poses and paths is not appropriate. Flexible assembly planning involves three aspects: task planning of the necessary steps and actions to achieve the overall goal/task; scheduling of these steps and actions; and motion planning for each step and action. Dual-arm manipulation further requires to decide about the allocation of task steps and actions to the individual arms. Moreover, the complexity of scheduling and motion planning is increased heavily, due to the necessity to closely coordinate the manipulators to prevent self-collisions of the robot. All four aspects – task planning, scheduling, allocation and motion planning – are closely interrelated and must be combined to achieve optimal plans with regard to some objective e.g., makespan. Henceforth, we refer to this instance of combined planning as an *assembly planning* problem.

#### 4.3.2 Integration Challenges

Obtaining an optimal solution to the assembly planning problem depends not only on the motion of the manipulators but also on the orders in which a workpiece is assembled, the components are taken from boxes or conveyor belts, processed by other machines, etc. These dependencies are all the more complex if connected systems or machines impose further temporal constraints. In addition, different assignments of sub-tasks to arms, while taking the individual working ranges into account as well as task steps in which the arms have to cooperate, lead to a further combinatorial complexity. The assembly planning problem was addressed in the literature *via* different methods, including prioritized TAMP (Kurosu et al., 2017), fixed-path planning (O’Donnell and Lozano-Pérez, 1989), and fixed-roadmap planning. For this paper, we analyze the latter approach, which uses a flexible model and solver for simultaneous task allocation and motion scheduling that is based on constraint programming (CP) and constraint optimization (Behrens et al., 2019a). The core modeling concepts was Ordered Visiting Constraints, which describe routine sequences of actions in production and time-scalable motion series. These are linked by so-called Connection Variables that act as the shared abstraction between the task and the motion models.

#### 4.3.3 Considerations

Four of our original questions are relevant in this application.

Q1: Similarly to the drill planning problem, we can keep each representation at its own level of abstraction, and employ a common language to pass relevant knowledge among those levels. Assembly planning for a large scale of items can possibly lead to a massive search space of mutually feasible solutions. However, industrial workplaces often have several characteristic properties that we can leverage to simplify the problem. For example in task modeling, it is safe to assume that many production routines can be described concisely by sequences of actions (e.g., drilling, picking, welding or joining) to perform with one of the robot arms at given locations, with temporal constraints and dependencies between them. This can be easily specified using Constraint Processing (CP) languages (Behrens et al., 2019a). Also, many industrial workplaces provide a controlled and unobstructed environment in which motions can be pre-computed in the form of time-scalable roadmaps. The obtained representation of motion is then discrete and ready to be connected to the high-level CP-based task model by some auxiliary variables so that it can be directly used by a constraint optimisation solver.

Q2: When we flatten out all levels of abstractions into one uniform level, or use an interface representation to manage interactions among abstraction levels, it then becomes straightforward to employ a dedicated solver that can read the uniform or the interface representation. In assembly planning, we can follow this logic, and employ a dedicated constraint optimisation solver for CP languages, e.g., Google Operation Research tools, to obtain an executable optimal assembly plan. The resulting plan is effectively a mutually feasible solution for all sub-problems: task planning, scheduling, allocation and motion planning.

Q3: Instead of directly specifying a sequence of production routines into a planning domain languages (e.g., a constraint problem), a multi-model learning method can be used for robot programming. In particular, a combination of learning from demonstration and requirements specification through natural language has been shown to be effective in preparing robot assembly planning domains for flexible manufacturing (Behrens et al., 2019b).

Q5: Industrial workplaces provide by design a controlled and unobstructed environment. Therefore, it can be assumed that all object locations and possible placements are known in advance, which allows for offline pre-calculation of motion roadmaps and a profile of potential collisions of the arms in motions. Furthermore, depending on the industrial setting, we may able to assume the absence of external interference, e.g., from humans.

### 4.4 Navigation Planning for Autonomous Underwater Vehicles

In this section, we focus on an instance of TAMP for Autonomous Underwater Vehicles (AUV) operating in spatially and temporally complex environments such as oceans. The problem analyzed in this section will be referred to as the *AUV mission planning* problem.

#### 4.4.1 Problem and Requirements.

AUVs are required to autonomously accomplish missions such as coverage or inspection of a sequence of regions in the ocean or sea. To perform such missions, an AUV must employ a mission planner that can reason about both high-level sequencing of the regions to be visited and low-level motions for navigating through them. While an AUV executes a series of tasks that can span over a period of several hours, the environment could change drastically due to the presence of tide and currents. An AUV mission planner should generate a combined task and motion plan that take into account not only the nonlinear dynamics of AUVs, natural obstacles in water, kinematic constraints, but also drift caused by the time-varying ocean currents. If these requirements, especially those imposed by dynamically changing environment, are not met, AUVs would attempt to carry out highly costly missions that are no longer feasible.

#### 4.4.2 Integration Challenges

In order to generate a feasible motion plan for an AUV, the intertwined dependencies between the tasks and dynamics of the AUVs and the environment should be considered in the initial planning phases. If the interactions with the environment are overlooked, it may be difficult or impossible to reach the regions of interest that the high-level task planner prescribes. Also, drift is usually modeled *via* a function whose inputs are position, depth, and time. Therefore, a particular ordering of visitation and position, for instance, could push the UAV further away from its goal because of drift. The AUV mission planning problem has been addressed in the literature by several different approaches. The one we analyze here builds a high-level navigation roadmap by sampling waypoints over the operational area and connecting neighboring waypoints to construct a network of navigation routes. This network avoids known obstacles, areas that are deemed too dangerous for the AUV, or other forbidden regions (McMahon and Plaku, 2016). The navigation roadmap is then combined with a Deterministic Finite Automaton (DFA) representing a regular language to compute sequences of waypoints that are compatible with the mission specification. This combined representation is then used to effectively guide a sampling-based motion planner that takes into account a model of the time-varying ocean currents in each its edge expansion.

#### 4.4.3 Considerations

For this application, we analyze two of our original questions.

Q1: As explained earlier, one way to deal with multiple levels of abstraction is to abstract away the continuous representation. In AUV mission planning, this particular choice would lead to discretising the relevant portion of the ocean in order to be able to impose high-level specifications for task and motion planning. It is problematic, however, to account for the nonlinear dynamics of the AUV and of the ocean currents in this discretisation. A complementary approach is to use a roadmap type of discretisation (i.e., a graph) which does not represent knowledge regarding the dynamics of the vehicle/environment, but contains knowledge about feasible states that satisfy the high-level task specification. An example of such a representation is roadmap coupled with a DFA (McMahon and Plaku, 2016). This is used as a guide to sample the continuous domain for obtaining a motion tree that is aware of constraints normally imposed on a continuous motion representation e.g., kinodynamic constraints or current drift.

Q3: The obvious candidate to leverage learning methods in this problem is to learn a drift model. In absence of reliable data to build predictive models of ocean currents, a simulator can take advantage of synthetic data derived from what is known of the physics of oceans.

### 4.5 Good Practices

One of the first questions that should be addressed when designing a TAMP solution for an application is to elicit the level of uncertainty inherent in the domain. Uncertainty manifests itself in many different ways: it may be relate to knowledge of goals, requiring them to be posted online, or it may relate to partial observability of the environment, the sudden appearance of obstacles, or uncertainty in the duration of motions. Understanding the nature of uncertainty at hand corresponds to answering questions Q4 and Q5. Analysing the types of uncertainty will narrow the range of task and motion planning algorithms that cater to that specific domain, and help in determining strategies to tackle the consequent challenges. For example, certain uncertainties can be completely hidden from the TAMP method and instead be dealt with during plan execution using existing control methods. Others, like the uncertain travel times in the multi-hauler planning application, should be considered explicitly in the design of a TAMP method, as disregarding them would violate important safety constraints. Sometimes, we can afford to totally ignore the presence of uncertainty, as in the case of the industrial manipulators operating in a controlled environment.

The next step in designing an appropriate TAMP method is to determine the levels of abstraction, and an effective method for their incorporation. This concerns the problem of finding one or more knowledge representation formalisms that are appropriate for expressing the requirements of the domain in question while affording efficient reasoning (addressing questions Q1 and Q2). Effectively dividing knowledge into different levels of abstraction is very challenging. In the drill planning application, for instance, a graph representation for the sequencing problem enables efficient high-level TSP computation while dedicating the geometric information to the motion planner. The TSP passes the knowledge of emerging obstacles to the motion planner, and the motion planner in response verifies the sequencing choice made by the TSP solver. Although this seems a reasonable distribution of knowledge between the two planners, the frequency of knowledge sharing between the two can be exponentially high. As this application shows, the question of how to interface several levels of abstraction is most crucial when there is high interdependency between the levels. On the contrary, in the multi-hauler planning, the interdependency is weak, and we can learn the low-level information and explicitly incorporate the learned model in the high-level task planner. In the latter, the question of how to interface efficiently is less crucial.

Another important issue that must be addressed in the early stages of designing an approach for combines TAMP is how to discretize the problem space. The right choice of discretization has a massive impact on the final solution. This directly relates to the size of the state space at the task level as well as the required calls to motion planning in loosely-coupled approaches like semantic attachments.

## 5 Summary and Outlook

The research questions we discussed above do not have simple answers. Depending on the domain at hand and the constraints of the application scenario, different answers may suit better for achieving an effective combined TAMP method. To aid the researcher or engineer in building a TAMP system, we have outlined an order in which questions can be approached, with the intention of reducing the amount of required backtracking in the decision making process.

As witnessed by the number of questions and the complexity of the overall topic, future research has the potential to simplify certain questions and maybe even eliminate certain trade-offs by providing more general solutions than we currently have at our disposal. Nevertheless, we conjecture that a one-fits-all method for solving combined TAMP will never exist, therefore the questions we have discussed in this article, as well as the proposed guidelines, will remain relevant in the future.
